# The Policy Dystopia Model: An Interpretive Analysis of Tobacco Industry Political Activity

**DOI:** 10.1371/journal.pmed.1002125

**Published:** 2016-09-20

**Authors:** Selda Ulucanlar, Gary J. Fooks, Anna B. Gilmore

**Affiliations:** 1 Department for Health and UK Centre for Tobacco and Alcohol Studies (UKCTAS), University of Bath, Bath, United Kingdom; 2 School of Languages and Social Sciences, Aston University, Birmingham, United Kingdom; San Diego State University, UNITED STATES

## Abstract

**Background:**

Tobacco industry interference has been identified as the greatest obstacle to the implementation of evidence-based measures to reduce tobacco use. Understanding and addressing industry interference in public health policy-making is therefore crucial. Existing conceptualisations of corporate political activity (CPA) are embedded in a business perspective and do not attend to CPA’s social and public health costs; most have not drawn on the unique resource represented by internal tobacco industry documents. Building on this literature, including systematic reviews, we develop a critically informed conceptual model of tobacco industry political activity.

**Methods and Findings:**

We thematically analysed published papers included in two systematic reviews examining tobacco industry influence on taxation and marketing of tobacco; we included 45 of 46 papers in the former category and 20 of 48 papers in the latter (*n* = 65). We used a grounded theory approach to build taxonomies of “discursive” (argument-based) and “instrumental” (action-based) industry strategies and from these devised the Policy Dystopia Model, which shows that the industry, working through different constituencies, constructs a metanarrative to argue that proposed policies will lead to a dysfunctional future of policy failure and widely dispersed adverse social and economic consequences. Simultaneously, it uses diverse, interlocking insider and outsider instrumental strategies to disseminate this narrative and enhance its persuasiveness in order to secure its preferred policy outcomes. Limitations are that many papers were historical (some dating back to the 1970s) and focused on high-income regions.

**Conclusions:**

The model provides an evidence-based, accessible way of understanding diverse corporate political strategies. It should enable public health actors and officials to preempt these strategies and develop realistic assessments of the industry’s claims.

## Abstract 2

In this thematic analysis, Selda Ulucanlar and colleagues develop taxonomies and an overall model to describe the political strategies used by the tobacco industry to influence policy around tobacco taxation and marketing.

## Author Summary

**Why Was This Study Done?:**

- Interference by the tobacco industry in government policy-making is known to be an important reason for governments’ failure to adopt proven measures to reduce tobacco consumption.
- Our study aimed to systematically review tobacco industry political activity from a critical societal perspective using in-depth empirical evidence.
- We set out to build a widely applicable model that governments in different countries could use to identify and preempt industry interference in policy.

**What Did the Researchers Do and Find?:**

- We analysed 65 papers included in previous systematic reviews that examined tobacco industry political activity in two policy areas: taxation and marketing of tobacco products.
- Using constructivist grounded theory, we identified industry arguments and techniques and then grouped these under the more general heading of strategies, finally developing a taxonomy and model of tobacco industry political activity.
- According to our Policy Dystopia Model, the industry produces an alarmist narrative that proposed policies will fail and lead to a great number of undesirable social and economic consequences (outlined in our taxonomy of discursive strategies).
- The industry also uses different methods (outlined in our taxonomy of instrumental strategies) to disseminate this narrative and persuade decision-makers in order to block policies.

**What Do These Findings Mean?:**

- Public health actors and policy-makers can use our model and taxonomies to better promote tobacco-related policies and to render ineffective tobacco industry opposition and political activity.
- In particular, they need to pay attention to alliance formation, information provision, interdisciplinary working, and transparency in policy-making.

## Introduction

Globally, tobacco kills 6 million people annually, potentially rising to 8 million by 2030 [1]. Of the 193 member states of the United Nations, 180 have now ratified the Framework Convention on Tobacco Control (FCTC), which outlines the evidence-based policies required to reduce tobacco use. The Convention has driven policy implementation internationally [2], but progress remains slow [3], with parties to the treaty identifying industry interference as the greatest impediment to progress [4]. It is increasingly recognised, therefore, that understanding, exposing, and addressing tobacco industry interference is key to progressing tobacco control [4,5]. The archive of more than 14 million internal tobacco industry documents disclosed as a result of litigation in the United States [6,7] has generated a unique evidence base for understanding the conduct of transnational tobacco companies (TTCs). However, given that there are now over 800 research publications based on these documents [8], evidence syntheses and conceptual models are required to more effectively use this evidence base to inform policy and augment social scientific understanding of TTC efforts to influence policy.

To date, only three systematic reviews—on TTC efforts to influence taxation [9], marketing [10], and policies in lower- and middle-income countries [11]—and two conceptual frameworks of tobacco industry political activity have been published [10,12]. In the latter category, only one was based on industry documents and used a systematic review of studies on marketing policy to begin to develop a taxonomy of tobacco industry political activity [10], drawing on Hillman and Hitt’s widely cited taxonomy of corporate political activity [13]. This work highlighted major shortcomings in Hillman and Hitt’s exchange-theory–based representation of corporate political activity as a mutually beneficial process through which corporate involvement in policy-making enables governments to develop optimal public policies.

Our current research builds on this initial work by incorporating evidence on the tobacco industry’s attempts to influence two key policy areas, taxation and marketing, and by taking a critical approach in order to develop a more comprehensive and grounded understanding of tobacco industry political activity. Our research questions were: What does the tobacco industry aim to achieve through its political activity? What would a critical taxonomy of tobacco industry political activity look like? By answering these questions, we also aimed to explore the value and limitations of Hillman and Hitt’s taxonomy in the context of the tobacco industry. We conducted in-depth interpretive analysis of papers included in two systematic reviews of tobacco industry political activity using grounded theory methods. Our analysis led to the development of two critical taxonomies that we hope will be of use to policy-makers and public health groups and an overall model that we present as an alternative conceptualisation of corporate political activity.

## Methods

Our data comprised the papers included in two systematic reviews that we had previously conducted on tobacco industry political activity, taxation [9], and marketing policies [10]. We based our analysis on these two systematic reviews, the methodological details of which are published elsewhere [9,10], because they provide the best quality of evidence in the area of inquiry. The two reviews used comprehensive searches (database searches, hand searches, internet searches, expert contact) to identify all relevant academic and grey literature, yielding 2,678 taxation and 1,754 marketing sources. Relevance and quality criteria were applied to identify the best quality evidence in the field; 46 and 48 papers were included, respectively. The database for the current analysis comprised 65 papers (Table 1). For taxation, we used all but one of 46 papers in the original review (we excluded the interim version of a report). For marketing, because this literature had only recently been reviewed by two of the same authors to develop a taxonomy [10], we selected half (24 of 48) of the papers in the review, using the following criteria: papers covering the last ten years (2003–2013) of the original review period and an even representation (within the constraints of the original sample) of geographic location and specific marketing policies. Four of the 24 were also in the taxation systematic review (with different sections analysed for each topic), making a total of 20 marketing papers. The included papers are listed in S1 Table (taxation) and S2 Table (marketing).

**Table 1:** The number of papers from the two systematic reviews included in the analysis by geographic location and topic.

Geographic location	Taxation* (total in systematic review: 46)	Marketing* (total in systematic review: 48)
N America/ Europe/Australasia	39	10
S America	-	3
Asia	5	4
Africa	1	-
Transnational	-	4
Total	45	20
Marketing and taxation duplicate papers	4	
Total** for taxonomy	65	

* Time period covered in systematic review—taxation: 1985–2010; marketing: 2003–2013.

** Total number of papers reaches only 65 rather than 69 because 4 papers featured in both the taxation and marketing systematic reviews, although different sections were analysed for the two topics.

Our approach to analysis was critical; drawing on the findings of our previous systematic review [10], we rejected Hillman and Hitt’s assumption that corporate political activity is a transparent and cooperative endeavour based on mutuality and honesty between public and private actors. We used Sarah Pralle’s concepts of expansion and containment of issues, actors, and spaces in advocacy work [14]. We also added the extra dimension of “voice” because we found that diverse voices were instrumental in framing, expanding, and containing issues and arguments. Additionally, we delineated stylistic features of tobacco industry political activity.

We used the techniques of constructivist grounded theory [15,16]: conceptual coding, systematic conceptual comparison, discourse sensitivity, attention to divergent data, and conceptual explanatory conclusions. Starting with the taxation literature, all the papers were entered into the ATLAS ti software and SU microcoded them for the smallest meaningful conceptual idea either as “argument” or “technique”; these were subsequently grouped under discursive and instrumental strategies, respectively. During this initial microcoding, we did not follow the coding schema of the two systematic reviews but conducted inductive and emergent coding, although it quickly became clear that most of the three strategies identified in Hillman and Hitt [12] and the three additional ones in Savell et al. [10] as well as the frames and arguments in the latter were relevant. However, we identified many new strategies and conceptually revised and refined others.

We included in the analysis all strategies identified in the dataset regardless of frequency but recorded the number of instances each was used (shown in our results tables). When the coding of the taxation papers was completed, a draft taxonomy was developed. Next, we inductively coded the marketing papers, regularly comparing the emergent coding frame with the draft taxonomy developed from the taxation papers and revising the latter as necessary. When all microcoding was completed, the taxonomy was reexamined for conceptual coherence and clarity and further modified. Next, a dynamic model was developed that accounted for not only the categories in the taxonomy but the relationships between them and the directions of influence. The study team (SU, GJF, ABG) met regularly (every 4–6 wk) throughout the study period to discuss microcodes, taxonomy categories, and the model, discussing and reaching consensus on divergent views.

## Results

### The Tobacco Industry’s Policy Aims

Our data showed that, faced with policy proposals aimed at reducing tobacco consumption, the tobacco industry attempts to secure a range of preferred outcomes that eliminate or limit the likely impact on its business (Table 2). Defeat—scrapping or shelving the policy—is the optimal outcome. Delay and weakening are sought if defeat is not possible. Foreclosing the legislative space is a future-facing strategy designed to make subsequent initiation and enactment of tobacco regulation more difficult. Once policy or regulation is in place, the industry may seek to overturn it. Alternatively, it attempts regulatory/policy avoidance through noncompliance, circumventing the rules or, for earmarked taxes, diverting earmarked funds.

**Table 2:** Tobacco industry preferred outcomes for tobacco control policies with examples.

**Defeat**
In Uzbekistan, British American Tobacco (BAT) succeeded in replacing planned tobacco control legislation with a voluntary advertising code (1994) [17].
A US state bill to raise cigarette tax, initially supported by the leadership of both the House and the Senate, was passed with a majority vote in the former but defeated in the latter after intense lobbying by the tobacco industry (1985) [18].
**Delay**
A proposed tax increase was blocked in a US state senate for almost a whole legislative session (1981) [19].
TTCs delayed the introduction of large pack health warnings in Canada by 11 months by invoking international trade agreements (1993) [20].
**Weakening**
Malaysia’s first comprehensive tobacco control legislation was watered down as a result of industry lobbying (1994) [21].
In Arizona, US, tobacco use prevention programmes were limited to narrow groups (e.g., young people, pregnant women), reducing their reach and effectiveness (1995) [22].
In Florida and Oregon, US, crippling amendments reduced the effectiveness of clean indoor air legislation and tobacco tax rises, respectively (1990; 1997) [23].
The industry voluntarily adopted weak European Economic Community health warnings to avoid stronger ones in Australia (1992) [24].
**Foreclosing**
Preemption: this was used in the US to restrict the legal authority of lower-level local jurisdictions to pass strong tobacco control laws once a bill was passed in the higher state legislature (1980s through 1990s) [25].
Agreement: a South Carolina, US, policy organisation in alliance with the tobacco industry secured pledges from legislators and public health groups not to seek tax rises or tobacco control policies (1993–1994) [26].
Infrastructural interference: Philip Morris sought to change state laws in Colorado, US, to make ballot initiation (for tobacco control bills) by the public more difficult (1994) [27].
Self-regulation: the industry sought voluntary marketing codes and youth education programmes to thwart effective regulation in many countries (1992–2005) [28].
**Overturning**
The tobacco industry worked for the passage of legislation to overturn previously enacted tobacco control legislation including tobacco-related Medicaid legislation in Florida 1999 [29].
In Lebanon, a decree banning advertisement was suspended after Philip Morris lobbied ministers (1980) [30].
**Avoidance**
Noncompliance: when entering the Czechoslovakia market, Philip Morris ignored preexisting advertising bans and placed large advertisements in public spaces (1993) [31].
Circumvention: when advertising was banned in Malaysia, TTCs set up companies for nontobacco products (e.g., fashion, music) in order to advertise indirectly using brand slogans and colours (1982) [21].
In Colorado, US, the industry lobbied the legislature to divert earmarked tax funds away from effective tobacco control policies to education and nursing services (2003) [27].

### Tobacco Industry Political Activity

Early on in our analysis, we recognised that industry political activity was performed through both arguments and actions and that these should be conceptualised as synergistic components of a dynamic model of political influence. We therefore distinguish between discursive (argument-based) and instrumental (action-based) strategies. We use the term “argument” as a subcategory of discursive strategies and the more neutral term “technique” instead of the often-used “tactic” as a subcategory of instrumental strategies. This terminology builds on that in our previous work [10]. We now present our taxonomies for each.

### Taxonomy of Discursive Strategies

The industry’s overall discursive strategy is to exaggerate—expand by argument—potential costs of proposed policy while simultaneously dismissing—containing by argument—potential benefits or denying these altogether. It seeks to build a comprehensive and credible narrative of undesirability for the policy by generating tailored arguments covering many different social domains (Table 3). Collectively, these mutually reinforcing arguments build the impression that the proposed public health policy will be detrimental to public health, the economy, and society. A key feature of this narrative is that it spans diverse sociopolitical domains and communities and is articulated not only by tobacco companies but also through a plurality of third-party voices, including those of law enforcer, concerned citizen, and public health policy analyst (Table 3). In this way, opposition to public health policies is represented not as the self-interested response of a profit-oriented business but the genuine concerns of different sectors of the public. A 1989 Philip Morris document explains:

“… we will need to talk in a variety of voices if what we want to say is to be heard, understood and acted upon. At times, we will speak as Philip Morris; sometimes we will need to speak as independent scientists, scientific groups and businessmen; and, finally, we will need to speak as the smoker.”

(Philip Morris document entitled “The ETS Battle” [32])

**Table 3:** Taxonomy of discursive strategies and arguments used to construct policy dystopia.

Discursive strategy	Domain		Argument	Voice	Instance in dataset
**Expanded/Created**					
**Unanticipated costs to economy and society**		The economy	Policy will lead to lost sales/jobs	Economist	[18], [24], [26], [30], [33], [34], [35], [36], [37], [38]
			Policy will lead to lost/unreliable tax revenue	Economist	[18], [25], [26], [33], [36], [39], [40], [41], [42], [43], [44]
		Law enforcement	Policy will increase illicit trade	Law enforcer	[18], [20], [26], [27], [33], [35], [39], [40], [42], [45], [46], [47], [48], [49]
			Policy will criminalise the public	Criminologist	[20], [35], [38], [50]
		The law	Breach of intellectual property laws	Corporate lawyer	[17], [20], [21], [24], [51], [52]
			Breach of trade agreements	Trade lawyer	[24], [38], [43]
			Public body acting beyond jurisdiction	Administrative lawyer	[20], [24], [40], [53]
		Politics/ Governance	Government is anti-free-enterprise	Concerned citizen/Business owner	[17], [18], [20], [21], [24], [25], [28], [30], [38], [40], [43], [50], [51], [52]
			Nanny state/slippery slope	Concerned citizen	[24], [26], [27], [28], [31], [36], [38], [39], [41], [52], [54]
			Government is unreasonable/unaccountable	Concerned citizen/Public ethicist	[18], [21], [22], [24], [26], [27], [28], [34], [35], [36], [37], [39], [41], [51], [52], [53], [54], [55], [56], [57], [58]
		Social justice	Policy is unfair to smokers	Public ethicist	[18], [19], [26], [27], [35], [36], [38], [40], [41], [42], [55], [57], [58], [59]
			Policy is regressive	Social reformer	[18], [19], [25], [33], [34], [35], [36], [39], [41], [54], [59], [60], [61], [62], [63]
**Unintended benefits to undeserving groups**			Smugglers will profit	Concerned citizen/Public ethicist	[27], [40], [45]
			Big business will profit	Concerned citizen/Public ethicist	[22], [41], [53], [55], [56], [64]
**Unintended costs to public health**			Policy will be counterproductive	Public health policy analyst	[20], [24], [25], [35], [36], [37], [38], [49], [51]
**Contained/Denied**					
**Intended public health benefits**		There is not (good) enough evidence		Scientist	[24], [37], [38], [51], [52], [65]
		Policy will not work		Public health policy analyst	[17], [21], [24], [28], [37], [38], [49], [62]
		Policy is not needed		Public health policy analyst	[17], [21], [24], [38], [66]
**Expected tobacco industry costs**		Policy will lead to reduced sales/jobs		Business owner	[17], [18], [19], [20], [25], [39], [42]
		Cost of compliance will be high		Business owner	[24], [37], [50]

#### Expanding and creating potential costs

The industry seeks to exaggerate [9,67–70] the costs of proposed policies using three related sets of arguments (Table 3). First, it expand*s* the types and reach of unanticipated costs to the economy and society and creates new costs relating to the economy, law enforcement, the law, politics and governance, and social justice. The breadth of arguments is wide-ranging. Key arguments include that public health policies will reduce sales and jobs in nontobacco sectors and that falling tobacco sales and increasing use of illicit tobacco will reduce tax revenues (economy). That tobacco control policies will increase illicit trade (law enforcement) is a well-used TTC argument. TTCs often claim that proposed policies are inconsistent with domestic or international trade and investment law, or that public bodies introducing them are committing procedural irregularities or acting beyond their legal remit, all of which will lead to expensive litigation (law). Tobacco control policies are projected as examples of bad governance and unjustifiable government interference in the market, incompatible with the organising philosophy of liberal democratic states (politics and governance), and smokers are represented as a “punished” minority whose civil rights are threatened (social justice). In this way and through early framing [64], the industry attempts to shift the debate away from the health effects of tobacco.

Second, it argues that unintended benefits will fall on undeserving groups (effectively a policy cost). For example, criminals will benefit from higher tobacco taxes, leading to increased illicit sales, or earmarked (hypothecated) tobacco taxes will result in undeserved extra income for already wealthy doctors, hospitals, and insurance companies. In these discourses, often delivered through third parties, big tobacco dissociates itself from big health care/big insurance and aligns itself instead with ordinary citizens.

Third, the industry argues that, far from introducing benefits, the policy will have unintended public health costs and contribute to increased smoking through a number of mediating psychosocial, behavioural, and economic mechanisms: more prominent pack health warnings will lead to “warning overload” so smokers will ignore them entirely; warnings and plain packaging will render cigarettes “forbidden fruit” and increase their attractiveness, especially to young people; plain packaging and increased illicit trade will force companies to compete on price, pushing prices down.

#### Containing and denying benefits

In addition, the industry seeks to contain or deny both the projected public health benefits of the policy and the costs to itself using a mutually reinforcing, interdependent set of arguments. While adverse impacts on other (more deserving) groups such as retailers, farmers, and advertisers are expanded, those on the tobacco industry are deemphasized. When such impacts are mentioned, they are presented not as a matter of private loss (of profits) to TTCs but public loss to the whole economy and society. In denying the public health benefits of policies, TTCs argue that the scientific evidence is inconclusive, for example because it relates to predicted—not actual—behaviour, that the policy will not work, or that it is simply not needed because, for example, the industry fulfils corresponding requirements through voluntary codes.

#### Absent benefits

Finally, wholly absent from the industry’s narrative are the wider benefits to the economy and society that commonly result from improvements in public health [71,72].

### Taxonomies of Instrumental Strategies

The industry uses both insider (legislative and governmental) and outsider (public domain) strategies [73,74] in order to persuade the public and decision-makers of the plausibility of its predictions encapsulated in the arguments outlined above (Table 4). Coalition management represents a key outsider strategy. The main insider strategy—“lobbying”—is not consistently defined in the literature, being used by US scholars to refer to the provision of information to policy-makers [13] and by others as a combination of information provision and pressure techniques [75]. We use the term in the non-US sense and further suggest that actions normally subsumed under “lobbying” can be disaggregated into the categories of information management and direct involvement/influence (Table 4), with information management straddling both insider and outsider domains as we explain below. Litigation and illicit trade are both outsider strategies.

**Table 4:** Taxonomy of instrumental strategies and techniques used.

Instrumental strategy		Technique		Instance in dataset
**Coalition management**		Constituency recruitment	Internal (tobacco companies and their staff)	[18], [20], [21], [23], [24], [25], [28], [33], [34], [37], [39], [40], [46], [51], [76], [77]
			External	[18], [19], [20], [21], [23], [24], [25], [26], [27], [28], [29], [30], [35], [36], [37], [41], [43], [44], [45], [49], [52], [58], [61], [62], [63], [65], [76], [77], [78], [79], [80], [81]
		Constituency fabrication		[18], [19], [24], [25], [26], [27], [31], [33], [35], [36], [40], [53], [55], [61], [62], [63], [71], [79], [82], [83]
		Constituency fragmentation		[18], [27], [37], [55], [56], [59], [77], [79], [80]
**Information management**	Production	Producing a skewed evidence base as corroboration for projected policy failure		[18], [19], [20], [21], [24], [25], [26], [27], [28], [30], [31], [33], [35], [37], [38], [39], [40], [41], [43], [44], [45], [46], [47], [49], [52], [59], [61], [62], [63], [64], [77], [79], [83]
		Intelligence gathering		[18], [19], [20], [21], [24], [26], [27], [35], [36], [37], [45], [52], [58], [64], [65], [70]
	Amplification	Wide dissemination of industry-sponsored information/evidence		[18], [20], [24], [25], [26], [27], [33], [34], [35], [36], [40], [41], [42], [43], [45], [48], [49], [52], [53], [55], [59], [61], [62], [64], [67], [77], [80]
		Disseminating misleading/confounding information		[22], [24], [26], [33], [34], [37], [41], [48], [53], [54], [55], [56], [58], [77]
	Suppression	Contesting/suppressing public health evidence		[17], [20],[24], [28], [30], [37], [38], [51], [52], [70]
		Silencing public health opponents		[24], [36]
	Credibility	Fronting: concealing industry links to information/evidence		[18], [22], [23], [24], [26], [27], [30], [33], [34], [37], [39], [40], [44], [52], [53], [55], [56], [59], [63], [64], [67], [77], [79], [82], [83]
	Reputation management	Rehabilitating industry reputation		[18], [20], [21], [37], [38], [45], [77]
		Discrediting public health advocates		[18], [20], [21], [22], [37], [38], [49], [67]
**Direct involvement and influence in policy**		Access		[17], [18], [19], [20], [21], [22], [23], [24], [25], [26], [27], [28], [31], [34], [36], [37], [39], [41], [43], [45], [46], [48], [52], [55], [56], [61], [62], [64], [65], [67], [71], [79], [80], [82], [84], [85]
		Incentives and threats		[18], [19], [20], [23], [24], [25], [37], [44], [52], [56], [64], [82], [83], [85]
		Actor in legislative processes		[18], [19], [20], [26], [31], [36], [39], [41], [43], [46], [47], [52], [56], [64]
		Actor in government decision-making		[17], [18], [20], [21], [24], [30], [31], [37], [46], [47], [52], [84], [86], [87]
**Litigation**		Legal action to contest/obstruct legislation/regulation		[20], [22], [27], [28], [41], [43], [44], [52], [57], [65], [83], [88], [89]
**Illicit trade**		Facilitating/conducting smuggling		[39], [45], [47], [49], [90]

#### Coalition management

TTCs build and manage coalitions to provide alternative and more credible platforms for their arguments using three techniques—constituency recruitment, fabrication, and fragmentation. Through constituency recruitment, TTCs form alliances with other tobacco companies, often in the form of national, regional, and international tobacco industry associations, and recruit their staff to take action on their behalf (internal constituency building). They also secure the support of an astonishing spectrum of social groups, from low-income workers to company executives and welfare reformers to pharmaceutical companies, creating “simulations of enthusiasm” [23] for its case (external constituency building).

We suggest that three forms of interest relationship underpin constituency building: “common interests,” which involve actors within the tobacco supply chain, such as other TTCs, tobacco growers, wholesalers, and distributors; “extended common interests” with other businesses whose interests can be tied—by arguments of varying degrees of validity—to tobacco sales, e.g., restaurants, bars, hotels, grocery shops, petrol stations, and packaging companies; and “grafted common interests” with organisations whose interests can only be linked to tobacco consumption through non-business arguments such as freedoms or tax equity and require strategic adoption of the organisations’ agendas. We have identified more than 50 instances of grafted common interest coalitions ranging from trade unions (opposition to excise taxes), women’s groups (domestic violence), and organisations representing ethnic minorities (equality and social justice) to legislative associations and diplomatic missions.

The most common recruitment mechanism is financial incentives (membership fees, contributions for projects, training, etc.), although nonmonetary support (e.g., joint project work) is also seen as important, rendering TTC support “impossible to replace with money alone” [80]. To avoid alienating recruited organisations, the industry uses intermediaries; for example, the US Tobacco Institute, the industry’s trade and lobbying organisation, set up a Labor Management Committee (LMC) in 1983 as a buffer between the industry and labour groups [61].

Under constituency fabrication, the industry uses preexisting legitimate and specially created organisations as well as individuals to act as front groups. Front groups remove the industry’s “fingerprints” [39] from information and evidence and allow it to fight its case without being “provocative, confrontational and counterproductive” [27]. A special kind of front group formation is “astroturfing” [91], in which otherwise disorganised citizens are sponsored and organised into visible and audible blocks of “grassroots” opposition. Astroturf organisations or fabricated constituencies can be created to oppose specific legislation or have a longer-term mission. The tobacco industry has operated through a large number of such organisations to further its political aims; our review has identified at least 15.

Once recruited, constituencies support the industry through lobbying (telephone calls, letters, meetings), media agenda-setting (editorial and advertisement placement), testifying at legislatures, and promoting self-regulation. The industry seeks to strengthen these effects by providing constituencies with media training and materials, information, petition templates, instructions on letter-writing, telephone numbers of politicians, and display materials.

Less commonly, the industry attempts to weaken or fragment (potentially) hostile constituencies (constituency fragmentation). For example, in order to preempt women being identified as “victims” of smoking and lending their support to tobacco control policies, it sought to “neutralize that threat” by co-opting women’s organisations into taking a neutral position to smoking [80] by supporting domestic violence campaigns. The industry also seeks to weaken tobacco control supporters through diverting and dissipating their efforts and resources, for example, creating a “flurry of legislative activity to confound the antis” [27] or supporting bills that the tobacco community has to fight at the same time as promoting the main proposed legislation.

#### Information management

The industry takes a comprehensive approach to information management, producing and widely disseminating favourable information and supressing and undermining information supportive of public health policies.

*Production*: To construct an alternative evidence base that substantiates its case, the tobacco industry produces information in a variety of formats (Table 5) on all aspects of proposed policies and potential (adverse) impacts, much of it inaccurate. TTCs determine much of the content and distribution of in-house and third-party produced information. Formal reports are channelled to governments and legislatures, but much original material is processed into easy-to-use campaign materials written in a plain and concise language of certainty. Scientific studies are often designed with in-built biases [24,48,83] to fashion findings consistent with industry interests, and where there is risk of findings unfavourable to the industry, studies are not conducted. The Tobacco Institute, for example, desisted from conducting studies on children’s smoking perceptions, as suggested by its public relations consultants, because of doubts over the likelihood of producing the desired results [68].

**Table 5:** Information management.

Sources/Producers of information	Types/Formats of information produced	Topics covered	Audiences/Users targeted
• Tobacco companies/industry associations • Nontobacco companies/business associations • Law firms • Accountancy firms • Consultancy/public relations firms • Market research organisations • Research organisations/think tanks • Public bodies • Nongovernmental/citizen organisations • Front organisations • Academics/universities	• Fact sheets/leaflets/booklets • Reports • Policy position papers • Briefing notes • Books • Polls/surveys • Studies conducted by consultancies • Scientific studies conducted by academics	• Economic/market baseline information • Impact of policies on the economy • Impact of policies on illicit tobacco trade • Impact of policies on consumer behaviour • Legality of policies/compliance with trade laws • Contribution of the tobacco industry to the economy	• Tobacco company staff • Ministers, government departments • Legislators • Recruited constituencies • Media/public

The industry also gathers intelligence by conducting surveys of public attitudes [24,27,58, 64], monitoring tobacco control activities and policies [18,27,37,52,68], and collecting information on politicians, tobacco control activists, and opinion leaders to determine their potential as allies or adversaries [19,20,21,37,63] in order to plan and target activities and to stymie regulation early on.

*Amplification*: TTCs, industry trade associations, recruited organisations, and front groups all cascade industry-favourable information and evidence to diverse audiences. Important TTC techniques are building long-term relations with media owners, managers, and journalists so they will “think again before publishing anti-industry propaganda” [27] and reducing the agenda-setting power of public health advocacy through competent use of the media: strategic timing, longer duration, higher intensity, broader coverage, and impact monitoring. Company and allied organisation websites provide access to anti-legislation campaigns and voting sites where fliers and handouts can be downloaded.

*Suppression*: Public relations firms, law firms, and co-opted academics are hired to develop and disseminate critiques of public health evidence, questioning its rigour and validity and undermining and diminishing its impact. TTCs also attempt to suppress the publication of public health evidence by, for example, seeking court injunctions on the grounds that the study is “misleading and deceptive” [24], restricting access of public health groups and politicians supportive of tobacco control measures to the media, and preventing journalists from covering unfavourable stories [24,30].

*Credibility*: TTCs distance themselves from the evidence they produce and fund to strengthen its credibility [27,60], for example, by failing to disclose funding, sponsorship, or authorship of reports and advertisements [59,61,83] and by using professional consultants who falsely claim independence [9,48] when delivering industry-controlled information and evidence.

*Reputation management*: TTCs and industry lobbyists attempt to represent the industry as a “good corporate citizen,” concerned about population welfare and the economy, at the same time as attempting to undermine the reputation of the public health community, as illustrated by a Philip Morris Long-Range Plan (1990–1992), which reported the company’s intention to “precisely identify, monitor, isolate and contest key individuals and organizations” [37].

#### Direct involvement and influence in policy

*Access*: The tobacco industry has built, over some decades, sophisticated routes into both legislatures and executive offices of governments, using a large pool of professional lobbyists, co-opted politicians, and public figures to engage in “dialogue” and to “discuss constructive solutions.” Although some of this contact occurs officially, as in the case of impact reports presented to officials, much of it is informal. In many instances, industry executives, professional consultants, lawyers, politicians, and public officials cohabit a small social world, and this expedites industry influence in a number of ways: multiple conflicting roles—an academic cardiologist serving as both a tobacco industry consultant and a scientific adviser to a country’s president [52]; a law firm simultaneously representing the tobacco industry, a major political party, and the medical association [81]; revolving door—a legislator becoming a Tobacco Institute lobbyist [36]; a high-ranking bureaucrat becoming a TTC president [20]; fortuitous links—encounters at social events; a TTC distributor’s business partner who happens to be the foreign minister [18]; a judge whose friend manages funds that include TTC stock [68].

*Incentives and threats*: Financial incentives have been offered to political parties, legislators, government ministers and officials, and candidates for political office and include contributions to political campaigns, entertainment, meals, travel, and leisure activities [18,19,21,23,25,31,52,63,85]. Threats with potentially far-reaching economic consequences are also used, covering disinvestment [17], withholding advertising revenue [37], taking legal action [20,25], and making large compensation claims [20].

*Actor in legislative processes*: Within legislatures, the industry has used two principal techniques: argument and procedural steering. Recruited legislators and third-party advocates rehearse and amplify the industry’s arguments in their testimonies. The chief US negotiator for the North American Free Trade Agreement, for example, was retained by two tobacco companies to tell the Canadian Commons Committee that plain packaging would be an “unlawful expropriation” of trademark rights and lead to staggering compensation claims [20]. Industry co-opted legislators can delay the passage of bills to the committee stage or steer proposed legislation to similarly co-opted committees where they are likely to be quashed.

*Actor in government decision-making*: TTCs have secured representation for themselves or their coalition partners on joint government committees and working groups established to devise or plan implementation of new regulation—even revenue allocation to tobacco control services. TTCs seek to take advantage of limited governance capacity, for example, in emerging markets to establish themselves as an indispensable source of expertise in public finance and tax regimes. A common approach is to establish insider status with senior politicians and civil servants and to use this to isolate and politically weaken departments charged with public health. For example, in Uzbekistan and Western Australia, health ministers were admonished by their (deputy) prime ministers for initiating policies on pack health warnings and advertising bans and asked to reverse their decisions following industry intervention [17,24].

#### Litigation

In addition to securing judgements that prevent laws from taking effect, legal action (in domestic courts or under trade and investment agreements) and the threat thereof help create “regulatory chill” by increasing the perceived costs of public health policies. Furthermore, claims of procedural illegality, for example, in the way public ballots are initiated or public health campaigns are funded, are used to frustrate these initiatives early on. From a strictly legal perspective, much litigation by the industry may appear ill-considered judging by the frequency of negative outcomes. But litigation or threat of litigation is used despite unfavourable legal opinion because the strategy is one facet of the industry’s construction of an alternative discursive reality, as illustrated in this BAT document:

“… even when arguments [relating to General Agreement on Tariffs and Trade (GATT) and Trade-Related Aspects of Intellectual Property Rights (TRIPS)] are sometimes not conclusive in themselves, they should be used uniquely to lobby local governments in our favour” [20].

#### Illicit trade

TTCs have a long history of involvement in the illicit tobacco trade [45,49,90]. Among other advantages, this enables their entry into new markets [47] and, importantly, provides “evidence” for the industry’s argument that tobacco control policies increase smuggling [39,45]. For example, R.J. Reynolds created both an anti-smuggling front group and Northern Brands International, which maintained the company’s smuggling networks in Canada and ultimately enabled the industry to use the problem of smuggling to reverse a tax rise [90].

## Discussion

**Fig 1:**
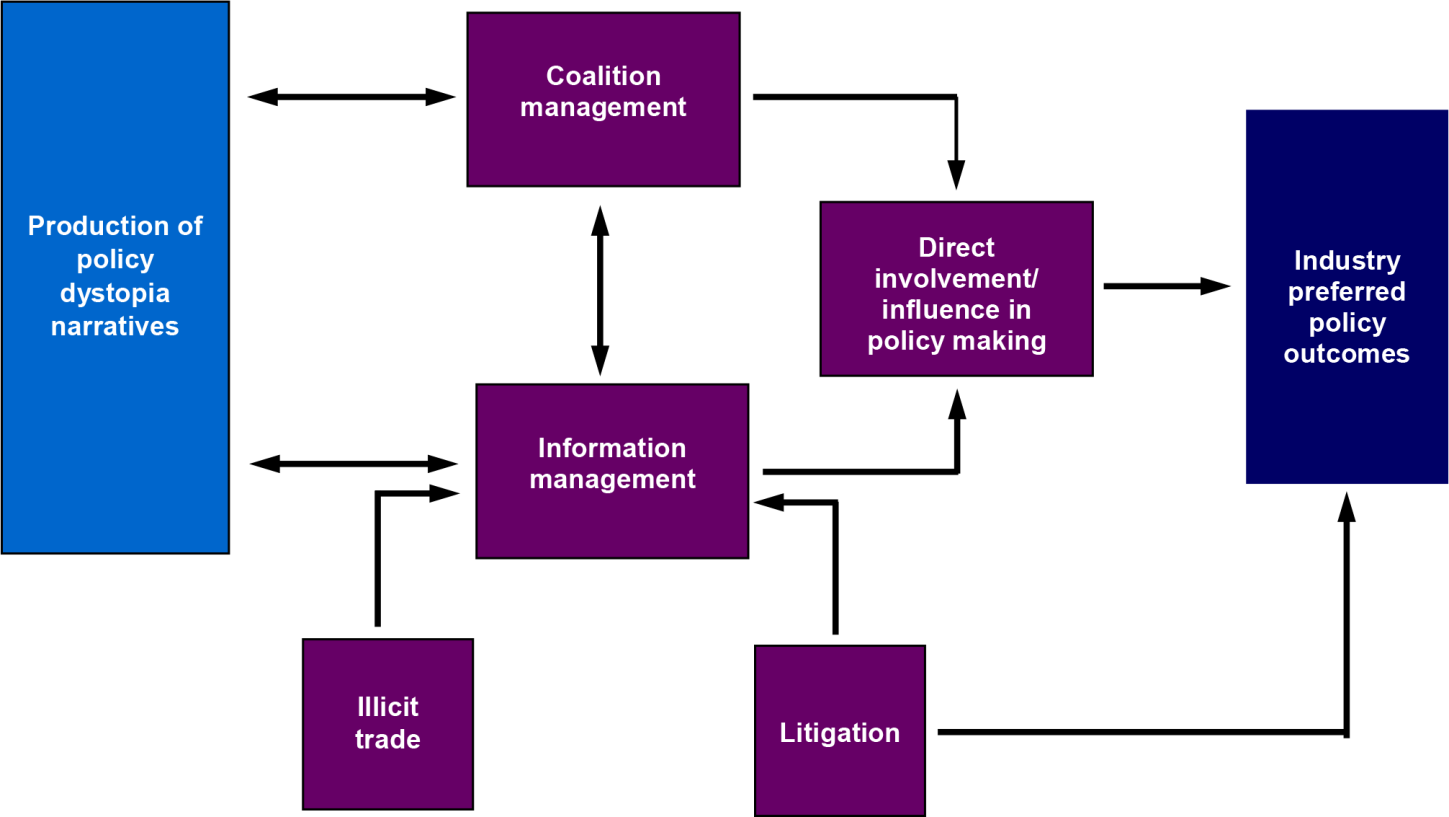
Policy Dystopia Model. Dystopian narratives (light blue box) are constructed and transmitted through instrumental strategies (purple boxes) to achieve preferred policy outcomes (dark blue box). The three key instrumental strategies, coalition management, information management, and direct involvement in decision-making, have recursive relations, reinforcing their effectiveness. Subsidiary strategies, illicit trade, and litigation feed into information management by increasing credibility of messages; litigation also directly impacts policy outcomes by stopping adoption or implementation of policies.

Our analysis suggests that the tobacco industry’s overall approach to opposing tobacco control policies is to construct an overarching narrative of a dysfunctional future that will ensue if the proposed policy is implemented and to widely disseminate this narrative in order to enhance its persuasiveness. We term this the Policy Dystopia Model. Embedded within a cost–benefit paradigm, the central narrative asserts that the policy will undermine public welfare because its costs will be large and will fall indiscriminately on a wide range of stakeholders, damaging the economy and society as a whole, while its benefits will be limited, non-existent, or enjoyed by the wrong stakeholders. This dystopian narrative is processed through three main (coalition management, information management, direct involvement/influence in policy) and two subsidiary (illicit trade, litigation) instrumental strategies (Fig 1). Within the model, there is fluidity and strong interdependence within and between discursive and instrumental strategies. For example, the strategies of information and coalition management facilitate each other: the support of a variety of constituencies allows diverse (tailored) dystopian arguments to be shaped and disseminated, and the voicing of these arguments by constituencies render them more credible and persuasive [91,92]. Furthermore, these diverse arguments help secure the support of a variety of groups whose interests might not otherwise align with those of the industry. Similarly, the strategy of facilitating or engaging in illicit trade feeds into the information strategy by providing “evidence” for the industry’s arguments concerning smuggling and reduced government revenues. The model represents a highly dynamic process in which different strategies are accentuated at a given time in line with prevailing political/economic contingencies.

The tobacco industry’s core premise is that policy-makers fail to consider, or underestimate, the potentially disastrous consequences of proposed public health policies, which, translated as costs, far outweigh any (marginal) benefits of the policy, making it unfeasible and damaging to legislators. To impress this alternative reality on the public and decision-makers and create an unwarranted impression of wide public and business support, it sets into motion an interlocking ensemble of activities: producing inaccurate and biased information, forming (unlikely) coalitions with diverse groups, and exerting direct influence on decision-makers.

The timeframe and geographic focus of the papers reviewed may limit the model’s applicability: although 32 out of 46 of the taxation papers and all the included marketing papers were published between 2003 and 2010, some covered historical events, and both were heavily dominated by North American/European/Australasian sources. However, our previous review and other work have shown that similar strategies are used in developed and developing economies and repeated over time [10,11], while the sociopolitical conditions in the emerging markets where the industry is currently establishing itself may not be that different from the historical contexts represented in our source literature [45–47]. Furthermore, it is apparent that TTCs are currently using the strategies and techniques we identify [5,93,94], although the weight given to different elements may vary over time. For example, the industry appears to be shifting the emphasis of its political strategies towards those that centre on illicit trade [68], litigation [95], and international trade agreements [96], with the use of third parties increasing in apparent response to the industry’s declining insider status [97,98]. Finally, we note that the tobacco industry uses similar strategies to oppose policies and regulation in areas other than marketing and taxation, such as smoke-free policies [11,98].

Our model and taxonomies take a critical perspective that recognises the fundamental conflict between corporate interests and public health. It was to address this conflict that the WHO FCTC’s Article 5.3 [99] and its implementing guidelines [100] suggested measures aimed at protecting public health policy-making from tobacco industry influence. A recent FCTC report [4] shows that much progress is still needed to address this interference. Our work, by identifying TTCs’ key strategies, can be used by public health advocates and policy-makers to direct efforts to effectively implement Article 5.3; it highlights, for example, the importance of full transparency. Second, on the basis that TTCs’ strategies and arguments are repeated over time and place, it can potentially be used to anticipate and counter industry opposition. For example, counterarguments and media strategies could be prepared in advance based on the details given above. Finally, the taxonomy can enable advocates and policy-makers to recognise and label contemporary industry strategies without using resources to investigate each incident.

Public policy formulation is a collective process, with corporations constituting only one part of an ensemble of governmental and nongovernmental actors and institutions. Whether corporations secure favourable policy outcomes depends not only on their actions but also on how those promoting the policy position their case and respond to corporate strategising, and how readily policy-makers accept corporations’ dystopian cost-based projections. Studies in other areas [101–103] indicate that governments attach considerable emphasis to corporate claims when contemplating health related policies. Furthermore, corporations’ emphasis on projected costs of public health policies dovetails with the “Better Regulation” agenda that increasingly dominates European, Australasian, and North American policy landscapes [104–106] and is often accompanied by mandatory impact assessments of costs and benefits. Both are expressions of prevailing neoliberal norms that posit state intervention in markets as inefficient and illiberal and promote minimal (“light-touch”) regulation of industries/business. Our analysis suggests that this convergence between corporate and government interests and the embedding of cost–benefit analysis within policy-making is likely to have major implications for public health. It raises the possibility of corporate annexation of public policy where corporate interests are better represented than broader public interests and where alternative approaches such as the precautionary principle [107] are squeezed out. A sustained public debate is needed on whether it is ethically defensible for the values of market economics, competition, and profit maximisation to guide deliberations on health- and welfare-oriented policies.

Both our previous work [10] and the wider analysis we offer here point to the inadequacy of exchange-based conceptualisations of corporate political activity as underpinning socially optimal policy-making, an approach encapsulated in the Hillman and Hitt taxonomy [13] and other work [108,109]. In the tobacco industry’s case, the failure of this theoretical approach to account for the industry’s political aims and actions as well as the costs of these for policy and public welfare have been demonstrated empirically. The industry has failed to act “responsibly” [110], systematically misused and misrepresented information and scientific evidence [69,111–113], exaggerated the costs of policies [94,114], obscured its involvement in the production of evidence designed to favour its case [114–117], and has been extensively involved in tobacco smuggling while opposing policies on the basis that they will increase illicit trade [39,90,118–122].

Although further research is needed to confirm this, our model and taxonomies are likely to be applicable to political action by nontobacco industries that also threaten the public’s health: for example, the oil and gas, ultra-processed food/soft drinks, and alcohol industries. There is growing evidence that nontobacco sectors use key strategies of the dystopia model in opposing public health policies: problem reframing and introducing “unintended” consequences [123]; exaggerating economic costs [124]; and constituency recruitment/fabrication and information management [125,126]. Our earlier work shows that the alcohol industry, for example, uses remarkably similar strategies to tobacco in opposing marketing policies [127]. Therefore, it is likely that management-focused theoretical positions are equally inadequate in studying and understanding political activity in other sectors. There is urgent need for more critical social science scholarship in this field.

## Conclusions

The Policy Dystopia Model and taxonomies can be a useful resource to the public health community and to policy-makers at national, regional, and international levels. First, this work will enable systematic focus on the types of dystopian narratives the industry is likely to produce for specific policies and the voices it is likely to use; these can then be preempted through effective counterarguments [128]. The health and societal benefits of the policies, in particular, need to be foregrounded to counter industry attempts to background them. Second, the taxonomies will enable health actors to anticipate and identify the kinds of coalitions the industry may attempt to build. Third, our work highlights that a crucial informational task for health actors is to deconstruct industry deconstructions of public science as well as to produce and disseminate information on the policy, paying attention to audience and language. Here, two strategies emerge as important: involving supportive organisations and individuals in producing and cascading information and enabling interdisciplinary networking by lawyers, public policy experts, economists, and scientists [129]. Fourth, the work highlights that government must ensure and the public health community must insist on transparency in official interactions with the industry and lobbyist access to legislatures as well as funding disclosure for all individuals and organisations acting as stakeholders. Finally, the model and taxonomies can place public health advocates and policy-makers in an advantageous position in which they are able to proactively plan narratives and strategies and not merely react to those of the industry. Further empirical work is required to examine whether the policy dystopia model is applicable to other tobacco control policy areas such as smoke-free environments as well as to nontobacco public health policies; how public and elected officials make sense of and respond to industry strategies; and what works in countering corporate political activity.

## Supporting Information

S1 Table Papers on taxation included in the analysis. (DOCX)

S2 Table Papers on marketing regulations included in the analysis. (DOCX)

## Abbreviations

BAT: British American Tobacco
CPA: corporate political activity
FCTC: Framework Convention on Tobacco Control
GATT: General Agreement on Tariffs and Trade
LMC: Labor Management Committee
TRIPS: Trade-Related Aspects of Intellectual Property Rights
TTC: transnational tobacco company
