# Supplementary material for: The Policy Dystopia Model: An Interpretive Analysis of Tobacco Industry Political Activity
Source: PLoS Med. 2016 Sep 20;13(9):e1002125. doi: 10.1371/journal.pmed.1002125 (PMC5029800; doi:10.1371/journal.pmed.1002125)
Supplement: S1 Table — (DOCX) [file pmed.1002125.s001.docx]

**S1 Table Papers on taxation included in the analysis**

| **Study** | **Geography** | **Policy that the tobacco industry attempted to influence** |
| --- | --- | --- |
| Alamar & Glantz, 2004  [1] | USA | Tax increases |
| Alamar et al, 2003  [2] | USA | Tax increases |
| Balbach & Campbell, 2009  [3] | USA | Tax increases |
| Balbach et al, 2006  [4] | USA | Tax increases  Tax funds for tobacco control |
| Balbach et al, 2000  [5] | USA | Tax increases |
| Barnes & Glantz, 2008  [6] | USA (Hawaii) | Tax increases  Tax structures |
| Begay et al, 1993  [7] | USA | Tax funds for tobacco control |
| Bialous & Glantz, 1999  [8] | USA | Tax increases  Tax funds for tobacco control |
| Breton et al, 2006  [9] | Canada | Tax reductions |
| Campbell & Balbach, 2008  [10] | USA | Tax increases |
| Campbell & Balbach, 2009  [11] | USA | Tax increases |
| Center for Tobacco Policy Research, 2008  [12] | USA | Tax increases |
| Dearlove & Glantz, 2000  [13] | USA | Tax increases |
| Epps-Johnson et al, 2009  [14] | USA | Tax increases |
| Gilmore & McKee, 2004  [15] | Former Soviet Union countries | Tax structures |
| Gilmore et al, 2005  [16] | Moldova | Tax structures |
| Gilmore et al, 2007  [17] | Uzbekistan | Tax reductions  Tax structures |
| Givel, 2006  [18] | USA | Tax increases |
| Givel, 2007  [19] | USA | Tax increases |
| Givel & Glantz, 2001  [20] | USA | Tax increases |
| Glantz & Balbach, 2000  [21] | USA | Tax increases |
| Goldman & Glantz, 1998  [22] | USA | Tax increases |
| Goldman & Glantz, 1999  [23] | USA | Tax increases  Tax funds for tobacco control |
| Hammond & White, 2001  [24] | Middle East | Tax increases |
| Heiser & Begay, 1997  [25] | USA | Tax increases |
| Hendlin et al, 2008  [26] | USA | Tax increases |
| Kelton & Givel, 2008  [27] | Canada | Tax increases |
| Koh, 1996  [28] | USA | Tax increases  Tax funds for tobacco control |
| Landman & Bialick, 2004  [29] | USA | Tax increases  Tax funds for tobacco control |
| Levenstein et al, 2005  [30] | USA | Tax increases |
| Lum & Glantz, 2008  [31] | USA | Tax increases  Tax funds for tobacco control |
| Lum et al, 2009  [32] | USA | Tax increases  Tax funds for tobacco control |
| Moon et al, 1993  [33] | USA | Tax increases |
| Morley et al, 2002  [34] | USA | Tax increases  Tax funds for tobacco control |
| Nakkash, 2007  [35] | Lebanon | Tax structures |
| Raebeck et al, 2009  [36] | USA | Tax increases |
| Spivak & Givel, 2005  [37] | USA | Tax increases  Tax funds for tobacco control |
| Stanton et al, 2009  [38] | USA | Tax increases  Tax funds for tobacco control |
| Sullivan et al, 2009  [39] | USA | Tax increases  Tax funds for tobacco control |
| Szilagyi & Chapman, 2003  [40] | Hungary | Tax increases  Tax structures |
| Torrijos & Glantz, 2005  [41] | USA | Tax increases  Tax funds for tobacco control |
| Traynor & Glantz, 1996  [42] | USA | Tax increases  Tax funds for tobacco control |
| Van Walbeek, 2003  [43] | South Africa | Tax increases |
| Welle et al, 2004  [44] | USA | Tax increases |
| Yerger & Malone, 2002  [45] | USA | Tax increases |

**References**

1. Alamar B, Glantz S. The tobacco industry s use of Wall Street analysts in shaping policy. Tob Control. 2004;13: 223–227.
2. Alamar B, Mahmoud L, Glantz SA. Cigarette Smuggling in California: Fact and Fiction, 2003. Center for Tobacco Control Research and Education. UC San Francisco: Center for Tobacco Control Research and Education. Available: <http://escholarship.org/uc/item/4fv0b2sz>
3. Balbach ED, Campbell RB. Union Women, the Tobacco Industry, and Excise Taxes A Lesson in Unintended Consequences. Am J Preventive Medicine. 2009;37: S121–S125.
4. Balbach ED, Herzberg A, Barbeau EM. Political coalitions and working women: how the tobacco industry built a relationship with the Coalition of Labor Union Women. J Epidemiol Commun H. 2006;60: 27–32.
5. Balbach ED, Traynor MP, Glantz SA. The implementation of California's tobacco tax initiative: The critical role of outsider strategies in protecting proposition 99. J Health Polit, Polic. 2000;25: 689–715.
6. Barnes RL, Glantz SA. Tobacco Control in Hawai'i: Progress in Paradise, 2008. Center for Tobacco Control Research and Education. UC San Francisco: Center for Tobacco Control Research and Education. Available: <http://escholarship.org/uc/item/6696s2c8>
7. Begay ME, Traynor M, Glantz SA. The Tobacco Industry, State-Politics, and Tobacco Education in California. Am J Public Health. 1993;83: 1214–1211.
8. Bialous SA, Glantz SA. Arizona's tobacco control initiative illustrates the need for continuing oversight by tobacco control advocates. Tob Control. 1999;8: 141-151.
9. Breton E, Richard L, Gagnon F, Jacques M, Bergeron P. Fighting a tobacco-tax rollback: A political analysis of the 1994 cigarette contraband crisis in Canada. J Public Health Pol. 2006;27: 77-99.
10. Campbell R, Balbach ED. Mobilising public opinion for the tobacco industry: the consumer tax alliance and excise taxes. Tob Control. 2008;17: 351–356.
11. Campbell RB, Balbach ED. Building alliances in unlikely places: Progressive allies and the tobacco institute's coalition strategy on cigarette excise taxes. Am J Public Health. 2009;99: 1188–1196.
12. Center for Tobacco Policy Research. Committee for a Healthy Future - Amendment 3: Tobacco Tax Campaign. Final evaluation report, 2008. St. Louis, MO: Washington University in St. Louis
13. Dearlove J, Glantz SA. Tobacco Industry Political Influence and Tobacco Policy Making in New York 1983-1999, 2000. Center for Tobacco Control Research and Education. UC San Francisco: Center for Tobacco Control Research and Education. Available: <http://escholarship.org/uc/item/2t45x412>
14. Epps-Johnson T, Barnes RL, Glantz SA. The Stars Aligned Over the Cornfields: Tobacco Industry Political Influence and Tobacco Policy Making in Iowa 1897-2009, 2009. Center for Tobacco Control Research and Education. UC San Francisco: Center for Tobacco Control Research and Education. Available: <http://escholarship.org/uc/item/5dt9w35k>
15. Gilmore A, McKee M. Moving East: how the transnational tobacco industry gained entry to the emerging markets of the former Soviet Union-part II: an overview of priorities and tactics used to establish a manufacturing presence. Tob Control. 2004;13: 151–160.
16. Gilmore AB, Radu-Loghin C, Zatushevski I, McKee M. Pushing up smoking incidence: plans for a privatised tobacco industry in Moldova. Lancet.2005; 365:1354–1359
17. Gilmore A, Collin J, Townsend J. Transnational tobacco company influence on tax policy during privatization of a state monopoly: British American Tobacco and Uzbekistan. Am J Public Health. 2007;97: 2001–2009.
18. Givel M. Punctuated Equilibrium in Limbo: The Tobacco Lobby and U.S. State Policymaking from 1990 to 2003. Policy Stud J. 2006;34: 405–418.
19. Givel M. Consent and countermobilization: the case of the National Smokers Alliance. J Health Commun. 2007;12: 339–358.
20. Givel MS, Glantz SA. Tobacco lobby political influence on US state legislatures in the 1990s. Tob Control. 2001;10: 124–134.
21. Glantz S, Balbach E. Tobacco war: inside the California battles. London: University of California Press; 2000.
22. Goldman LK, Glantz SA. Tobacco Industry Political Expenditures and Tobacco Policy Making in Oregon: 1985-1997, 1998. Center for Tobacco Control Research and Education. UC San Francisco: Center for Tobacco Control Research and Education. Available: <http://escholarship.org/uc/item/7r2969ks>
23. Goldman LK, Glantz SA. The passage and initial implementation of Oregon's Measure 44. Tob Control 1999;8: 311-322. doi:10.1136/tc.8.3.311
24. Hammond R, White CM. Voices of Truth, vol. 2: Multinational Tobacco Industry Activity in the Middle East: A review of Internal Industry Documents, 2001. Tobacco Control. UC San Francisco: Center for Tobacco Control Research and Education. Available: <https://escholarship.org/uc/item/1v6935q0>
25. Heiser PF, Begay ME. The campaign to raise the tobacco tax in Massachusetts. Am J Public Health. 1997;87: 968–973.
26. Hendlin YH, Barnes R, Glantz SA. Tobacco Control in Transition: Public Support and Governmental Disarray in Arizona 1997-2007, 2008. Center for Tobacco Control Research and Education. UC San Francisco: Center for Tobacco Control Research and Education. Available: <http://escholarship.org/uc/item/1gh7g5p1>
27. Kelton JMH, Givel MS. Public policy implications of tobacco industry smuggling through native American reservations into Canada. Int J Health Serv. 2008;38: 471–487.
28. Koh HK. An analysis of the successful 1992 Massachusetts tobacco tax initiative. Tob Control. 1996;5: 220–225.
29. Landman A, Bialick P. Tobacco Industry Involvement in Colorado. Tobacco Control, 2004. UC San Francisco: Center for Tobacco Control Research and Education. Available: <http://escholarship.org/uc/item/9d880448>
30. Levenstein C, Delaurier GF, Ahmed S, Balbach ED. Labor and the tobacco institute's labor management committee in new york state: the rise and fall of a political coalition. NS. 2005;15(2): 135-152.
31. Lum K, Glantz SA. The Defeat of Oregon's Tobacco Tax Initiative in 2007, 2008. Center for Tobacco Control Research and Education. UC San Francisco: Center for Tobacco Control Research and Education. Available: <http://escholarship.org/uc/item/6kt451hp>
32. Lum KL, Barnes RL, Glantz SA. Enacting tobacco taxes by direct popular vote in the United States: lessons from 20 years of experience. Tob Control. 2009;18: 377–386.
33. Moon RW, Males MA, Nelson DE. The 1990 Montana Initiative to Increase Cigarette Taxes: Lessons for Other States and Localities. J Public Health Pol. 1993;14: 19–33.
34. Morley CP, Cummings KM, Hyland A, Giovino GA, Horan JK. Tobacco Institute lobbying at the state and local levels of government in the 1990s. Tob Control. 2002;11: I102–I109.
35. Nakkash R. Tobacco industry strategies in Lebanon: an analysis of internal tobacco industry documents. PhD Thesis. London School of Hygiene and Tropical Medicine. 2007.
36. Raebeck A, Campbell R, Balbach E. Unhealthy Partnerships: The Tobacco Industry and African American and Latino Labor Organizations. J Immigr Minor Health. 2010;12: 228-233. doi: 10.1007/s10903-009-9269-0.
37. Spivak A, Givel MS. From Industry Dominance to Legislative Progress: The Political and Public Health Struggle of Tobacco Control on Oklahoma, 2005. Tobacco Control. UC San Francisco: Center for Tobacco Control Research and Education. Available: <http://escholarship.org/uc/item/65d3s7qb>
38. Stanton C, Barnes R, Glantz SA. Tobacco Control in Maine, 1979–2009: The Power of Strategic Collaboration, 2009. Available: <http://tobacco.ucsf.edu/states>
39. Sullivan S, Barnes RL, Glantz SA. Shifting Attitudes Towards Tobacco Control in Tobacco Country: Tobacco Industry Political Influence and Tobacco Policy Making in South Carolina. 2009. Center for Tobacco Control Research and Education. UC San Francisco: Center for Tobacco Control Research and Education. Available: <http://escholarship.org/uc/item/278790h5>
40. Szilagyi T, Chapman S. Tobacco industry efforts to keep cigarettes affordable: a case study from Hungary. Cent Eur J Public Health. 2003;11: 223–228.
41. Torrijos R, Glantz SA. Tobacco Control Policy Making in Montana 1979-2005: Falling Off the Horse at the Finish Line, 2005. Center for Tobacco Control Research and Education. UC San Francisco: Center for Tobacco Control Research and Education. Available: <http://escholarship.org/uc/item/2rj5f9cr>
42. Traynor MP, Glantz SA. California's tobacco tax initiative: The development and passage of Proposition 99. J Health Polit Polic. 1996;21(3): 543-585.
43. van Walbeek C. Tobacco Excise Taxation in South Africa. San Francisco: WHO Tobacco Control Papers, 2003. Center for Tobacco Control Research and Education UCSF. Available: <https://escholarship.org/uc/item/4x68v8f8#page-3>
44. Welle J, Ibrahim JK, Glantz SA. Tobacco Control Policy Making in North Dakota: A Tradition of Activism, 2004. Center for Tobacco Control Research and Education. UC San Francisco: Center for Tobacco Control Research and Education. Available: <http://escholarship.org/uc/item/9v58x8ps>
45. Yerger VB, Malone RE. African American leadership groups: smoking with the enemy. Tob Control. 2002;11: 336–345.
