# Supplementary material for: The Policy Dystopia Model: An Interpretive Analysis of Tobacco Industry Political Activity
Source: PLoS Med. 2016 Sep 20;13(9):e1002125. doi: 10.1371/journal.pmed.1002125 (PMC5029800; doi:10.1371/journal.pmed.1002125)
Supplement: S2 Table — (DOCX) [file pmed.1002125.s002.docx]

**S2 Table Papers on marketing regulations included in the analysis**

| **Study** | **Geography** | **Policy that the tobacco industry attempted to influence** |
| --- | --- | --- |
| Apollonio & Malone, 2010  [1] | USA | Youth access regulations |
| Assunta & Chapman, 2004  [2] | Malaysia | Regulating promotion/  Health warnings |
| Chapman & Carter, 2003  [3] | Australia | Health warnings |
| Epps-Johnson et al, 2009  [4] | USA | Vending machines |
| Freeman et al, 2008  [5] | Transnational | Standardised packaging |
| Gilmore et al, 2006  [6] | Uzbekistan | Advertising/  Health warnings |
| Hammond, 2010  [7] | Transnational | Standardised packaging |
| Hiilamo et al, 2012  [8] | Transnational | Health warnings |
| Hoek et al, 2012  [9] | New Zealand | Retail displays |
| Kennedy et al, 2011  [10] | USA | Product placement |
| Ling et al, 2010  [11] | USA | Sport sponsorship |
| Mackey et al, 2013  [12] | Uruguay | Standardised packaging |
| McDaniel & Malone, 2009  [13] | USA | Product descriptors on packs/  Advertising/  Direct marketing |
| Nakkash & Lee, 2009  [14] | Lebanon | Marketing restrictions/  Health warnings |
| Outterson, 2011  [15] | USA | Health warnings |
| Physicians for Smoke-Free Canada, 2008  [16] | Transnational (mostly Canada) | Standardised packaging/ Health warnings/  Advertising |
| **Study**  **[**] | **Geography** | **Policy that the tobacco industry attempted to influence** |
| Sebrie et al, 2005  [17] | Argentina | General marketing |
| Sebrie & Glantz, 2007  [18] | Latin America | General marketing |
| Shirane et al, 2012  [19] | Czech Republic | Advertising |
| Stanton et al, 2009  [20] | USA | Displays/  Sales to children |
| Szilagyi & Chapman, 2004  [21] | Hungary | Advertising |
| Washington et al, 2012  [22] | USA | Advertising |
| Welle et al, 2004  [23] | USA | Vending machines |
| WHO (World Health Organisation), 2008  [24] | Middle East (UAE & Lebanon) | Advertising/  Promotion |

**References**

1. Apollonio DE, Malone RE. The “We Card” Program: Tobacco Industry “Youth Smoking Prevention” as Industry Self-Preservation. Am J Public Health.2010;100: 1188–1201. doi: 10.2105/ajph.2009.169573
2. Assunta M, Chapman S. A mire of highly subjective and ineffective voluntary guidelines: tobacco industry efforts to thwart tobacco control in Malaysia. Tob Control. 2004;13: 43-50.
3. Chapman S, Carter M. "Avoid health warnings on all tobacco products just as long as we can": a history of Australian tobacco industry efforts to avoid, delay and dilute health warnings on cigarettes. Tob Control. 2003;12: 13-22.
4. Epps-Johnson T, Barnes RL, Glantz SA. The Stars Aligned Over the Cornfields: Tobacco Industry Political Influence and Tobacco Policy Making in Iowa 1897-2009, 2009. Center for Tobacco Control Research and Education. UC San Francisco: Center for Tobacco Control Research and Education. Available: <http://escholarship.org/uc/item/5dt9w35k>
5. Freeman B, Chapman S, Rimmer M. The case for the plain packaging of tobacco products. Addiction. 2008;103: 580–590. doi: 10.1111/j.1360-0443.2008.02145.x
6. Gilmore AB, Collin J, McKee M. British American tobacco's erosion of health legislation in Uzbekistan. BMJ. 2006;332(7537): 355-358.
7. Hammond D. “Plain packaging” regulations for tobacco products: the impact of standardizing the color and design of cigarette packs. Salud Pública de México. 2010. 52: S226–S232. doi: 10.1590/s0036-36342010000800018
8. Hiilamo H, Crosbie E, Glantz SA. The evolution of health warning labels on cigarette packs: the role of precedents, and tobacco industry strategies to block diffusion. Tob Control. 2012. doi:10.1136/tobaccocontrol-2012-050541.
9. Hoek J, Vaudrey R, Gendall P, Edwards R, Thomson G. Tobacco retail displays: a comparison of industry arguments and retailers’ experiences. Tob Control. 2012;21: 497-501. doi: 10.1136/tc.2011.043687
10. Kennedy A, Sullivan S, Hendlin Y, Barnes R, Glantz SA. Tobacco Control in Florida 1999–2011: The Good, The Bad, and The Ugly, 2011. Available: <http://tobacco.ucsf.edu/states>
11. Ling PM, Haber LA, Wedl S. Branding the Rodeo: A Case Study of Tobacco Sports Sponsorship. Am J Public Health. 2010;100: 32–41. doi: 10.2105/ajph.2008.144097
12. Mackey TK, Liang BA, Novotny TE. Evolution of Tobacco Labelling and Packaging: International Legal Considerations and Health Governance. Am J Public Health. 2013;103(4): e39-43 doi: 10.2105/AJPH.2012.301029.
13. McDaniel PA, Malone RE. Creating the “Desired Mindset”: Philip Morris’s Efforts to Improve Its Corporate Image Among Women. Women Health .2009;49: 441–474. doi: 10.1080/03630240903238800
14. Nakkash R, Lee K. The tobacco industry’s thwarting of marketing restrictions and health warnings in Lebanon. Tob Control. 2009;18: 310–316. doi: 10.1136/tc.2008.029405
15. Outterson K. Smoking and the First Amendment. New Eng J Med. 2011;365: 2351–2353. doi: 10.1056/nejmp1113011
16. Physicians for Smoke-free Canada. Plot against plain packaging, 2008. Ottawa, Ontario. Available: <http://www.smoke-free.ca/pdf_1/plotagainstplainpackaging-apr1%27.pdf>
17. Sebrie EM, Barnoya J, Perez-Stable EJ, Glantz SA. Tobacco industry successfully prevented tobacco control legislation in Argentina. Tob Control. 2005;14: e2 doi: 10.1136/tc.2005.011130.
18. Sebrie EM, Glantz SA. Tobacco industry “Youth smoking prevention” programs to undermine meaningful tobacco control in Latin America. Am J Public Health. 2007;97: 1357–1367. doi: 10.2105/ajph.2006.094128
19. Shirane R, Smith K, Ross H, Silver KE, Williams S, et al. Tobacco Industry Manipulation of Tobacco Excise and Tobacco Advertising Policies in the Czech Republic: An Analysis of Tobacco Industry Documents. PLoS Medicine. 2012;9: e1001248. doi: 10.1371/journal.pmed.1001248
20. Stanton C, Barnes R, Glantz SA. Tobacco Control in Maine, 1979–2009: The Power of Strategic Collaboration, 2009. Available: <http://tobacco.ucsf.edu/states>
21. Szilagyi T, Chapman S. Tobacco industry efforts to erode tobacco advertising controls in Hungary. Cent Eur J Public Health. 2004;12: 190–196.
22. Washington MD, Barnes RL, Glantz SA. Good Start Out of the Gate: Tobacco Industry Political Influence and Tobacco Policymaking in Kentucky 1936-2012, 2012. UC San Francisco: Center for Tobacco Control Research and Education. Available: <http://escholarship.org/uc/item/10k3p8m5>
23. Welle J, Ibrahim JK, Glantz SA. Tobacco Control Policy Making in North Dakota: A Tradition of Activism, 2004. Center for Tobacco Control Research and Education. UC San Francisco: Center for Tobacco Control Research and Education. Available: <http://escholarship.org/uc/item/9v58x8ps>
24. WHO. Voice of Truth. Egypt, 2008. WHO Regional Office for the Eastern Mediterranean. Available: <http://applications.emro.who.int/dsaf/dsa910.pdf>
